# Editorial: The Role of the EMT Program in Regulating the Immune Response in Carcinoma

**DOI:** 10.3389/fimmu.2022.940164

**Published:** 2022-05-30

**Authors:** Anushka Dongre, Sandra Ortiz-Cuaran, Hasan Korkaya

**Affiliations:** ^1^ Department of Biomedical Sciences, Cornell University, Ithaca, NY, United States; ^2^ Univ Lyon, Claude Bernard Lyon 1 University, INSERM 1052, CNRS 5286, Centre Léon Bérard, Cancer Research Center of Lyon, Lyon, France; ^3^ Georgia Cancer Center, Augusta University, Augusta, GA, United States

**Keywords:** epithelial- mesenchymal transition (EMT), immune checkpoint inhibitors, immune suppresion, tumor microenvironment, cancer progression

The Epithelial-to-Mesenchymal Transition (EMT) is a cell-biological process that operates during embryonic development, wound-healing, and carcinoma progression. During this process, cells typically shed the expression of epithelial markers, such as E-Cadherin, and express instead, mesenchymal markers, such as vimentin, fibronectin and certain master EMT-inducing transcription factors (EMT-TFs), notably Zeb1, Twist, Snail and Slug. This is a highly dynamic process that often gives rise to a series of intermediate phenotypic states arrayed along the epithelial-mesenchymal polarization spectrum (1, 2). While the EMT program has long been known to enhance metastatic potential and resistance to chemotherapies and targeted therapies, its ability to regulate the immune response and promote resistance to anti-tumor immunity has only recently been uncovered (3, 4).

Immunotherapies in general, and checkpoint blockade inhibitors in particular, have led to durable responses and prolonged survival in patients whose tumors failed to respond to conventional therapies. However, while immunotherapies have had unprecedented success in some patients with certain cancers such as melanoma and non-small cell lung cancer, a number of other malignancies have shown little or no response. This has generated the need to identify novel therapeutic vulnerabilities, which can be targeted in combination with known drivers of resistance to enhance the efficacy of immunotherapies. The ability of the EMT program to regulate the immune response brings to the forefront the possibility of targeting this tumor cell-intrinsic process to enhance anti-tumor immunity and subsequent responses to many forms of therapy. In this particular Research Topic, we present a collection of reviews and original research articles that document multiple mechanisms by which the EMT program regulates the immune response.

A few studies have demonstrated the ability of the EMT program to promote immune-evasive and immune-suppressive features in carcinomas. An unexplored avenue has been the universal applicability of the EMT program in majority of solid tumors to modulate the immune response in multiple other cancer types. Along these lines, Benboubker et al., discuss in their review how the phenotypic plasticity of melanomas promotes immune escape. They document several studies that demonstrate how de-differentiated melanoma cells with more-mesenchymal properties evade immune attack by the loss of melanoma antigens. Moreover, over-expression of Zeb1 in melanoma cells dampens their ability to release CXCL10, a potent chemoattractant for CD8^+^ T-cells. As a consequence, Zeb1 expressing melanoma cells exclude CD8^+^ T-cells and are resistant to anti-PD1 immune checkpoint blockade. Meng et al., focus specifically on uveal melanoma and demonstrate that the transcription factor *PRRX1* promotes uveal melanoma progression by activating the EMT program in these cells. Strikingly, in addition to promoting invasion and motility, the expression of *PRRX1* was associated with the expression of multiple inhibitory immune checkpoint molecules and poor prognosis.

Further elaborating on the broad spectrum of EMT-induced immune-suppression, Gu et al., address the acquisition of immune-evasive and immune-suppressive properties by soft tissue sarcomas, which are also enriched in cancer stem-like cells. By using a combination of machine learning and transcriptomic approaches, the authors identified stemness signatures in patient samples. Importantly, a low stemness signature was associated with increased activation of various innate and adaptive immune cells, better prognosis and an increased likelihood to respond to immunotherapy.

As mentioned above, the EMT program is a highly dynamic process with multiple intermediate or hybrid states that can also exist along the polarization spectrum. Sahoo et al., focused on determining whether carcinoma cells residing in a hybrid epithelial-mesenchymal state also exhibit immune-evasive features. To this end, the authors constructed and simulated the dynamics of a minimalistic regulatory network that contained hallmark EMT markers and PD-L1 (Programmed death-ligand 1). The simulation network, which was also integrated with scRNA-Seq and bulk RNA-Seq, revealed that cancer cells residing in a hybrid EMT state are indeed likely to express higher levels of PD-L1. Moreover, in addition to expressing PD-L1, breast cancer cells that exhibit a hybrid EMT state showed enhanced resistance to anti-estrogen therapy.

In terms of clinical utility, Zhao et al., review recent literature that is centered on targeting the hepatocyte growth factor/cellular-mesenchymal-epithelial transition (HGF/c-Met) signaling pathway in hepatocellular carcinomas. The authors summarize multiple clinical studies that demonstrate the potent anti-tumor activity of a MET inhibitor and suggest targeting MET in combination with other biomarkers as a therapeutic strategy for patients with advanced hepatocellular carcinoma. Additionally, Engelsen et al., review emerging evidence supporting the utility of targeting the receptor tyrosine kinase, AXL, to enhance the efficacy of immune checkpoint blockade inhibitors. While AXL is expressed by multiple cell types, in the context of cancer cells, it is closely associated with epithelial plasticity. Strikingly, its expression is induced on cells undergoing an EMT as well as on cells with high stem cell-like phenotype. Moreover, targeting AXL can induce profound changes within the tumor microenvironment, likely enhancing sensitization to immune checkpoint blockade inhibitors.

We hope that this special Research Topic collection contributes to further strengthening the association of the EMT program with immune-suppression in multiple cancer types, making it an attractive therapeutic target and predictive criteria to consider while implementing immunotherapeutic strategies.

## Author Contributions

All authors listed have made a substantial, direct, and intellectual contribution to the work and approved it for publication.

## Conflict of Interest

The authors declare that the research was conducted in the absence of any commercial or financial relationships that could be construed as a potential conflict of interest.

## Publisher’s Note

All claims expressed in this article are solely those of the authors and do not necessarily represent those of their affiliated organizations, or those of the publisher, the editors and the reviewers. Any product that may be evaluated in this article, or claim that may be made by its manufacturer, is not guaranteed or endorsed by the publisher.

## References

[1] Aiello NM Maddipati R Norgard RJ Balli D Li J Yuan S. EMT Subtype Influences Epithelial Plasticity and Mode of Cell Migration. Dev Cell (2018) 45:681–695 e684. doi: 10.1016/j.devcel.2018.05.027 29920274 PMC6014628

[2] Lambert AW Pattabiraman DR Weinberg RA. Emerging Biological Principles of Metastasis. Cell (2017) 168:670–91. doi: 10.1016/j.cell.2016.11.037 PMC5308465 28187288

[3] Terry S Savagner P Ortiz-Cuaran S Mahjoubi L Saintigny P Thiery JP. New Insights Into the Role of EMT in Tumor Immune Escape. Mol Oncol (2017) 11:824–46. doi: 10.1002/1878-0261.12093 PMC5496499 28614624

[4] Dongre A Weinberg RA. New Insights Into the Mechanisms of Epithelial-Mesenchymal Transition and Implications for Cancer. Nat Rev Mol Cell Biol (2019) 20:69–84. doi: 10.1038/s41580-018-0080-4 30459476

